# Research on the accelerating effect and driving mechanism of the African swine fever epidemic on the capitalization of hog farming: evidence from China

**DOI:** 10.3389/fvets.2025.1690105

**Published:** 2025-10-24

**Authors:** Junguo Hua, Meng Tian, Yunfei Jia, Yufan Chen

**Affiliations:** ^1^College of Economics and Management, Henan Agricultural University, Zhengzhou, China; ^2^Institute of Animal Science, Chinese Academy of Agricultural Sciences, Beijing, China

**Keywords:** hog industry, African swine fever, capitalization of hog farming, industrial agglomeration, government support

## Abstract

**Introduction:**

The outbreak of African swine fever (ASF) has led to a sharp increase in the price of hogs, accelerating the capitalization process of the hog industry and causing China’s hog industry to enter a “super hog cycle,” which has attracted much attention.

**Methods:**

Based on this, to clarify the mechanism of ASF on the capitalization of hog farming and enrich the relevant theoretical research, this paper uses panel data from 30 provinces in China from 2013 to 2023, and adopts the difference-in-differences model (DID) and the moderation effect model to analyze the impact of the epidemic outbreak on the capitalization of pig farming, providing a new perspective for exploring the influencing factors of hog farming capitalization.

**Results and discussion:**

The research found that ASF significantly accelerated the capitalization process of hog farming. This conclusion remained valid after undergoing a series of robustness tests. Moreover, the improvement of industrial agglomeration degree and the strengthening of government support would have a significant positive regulatory effect on it. Heterogeneity analysis revealed that in regions with high agricultural capital stock and non-feed-producing areas, the positive impact of ASF on the capitalization process of hog farming was more significant. Based on this, the article proposed policy suggestions such as strengthening the epidemic prevention and control system, preventing excessive expansion of hog production capacity, coordinating efforts through multiple channels to enhance the capitalization level of the hog industry, and promoting the high-quality development of the hog industry.

## 1 Introduction

China is a major producer and consumer of pork, and the hog industry plays a pivotal role in safeguarding national food security, increasing farmers’ income, and promoting rural economic development. However, since the beginning of the 21st century, frequent outbreaks of swine diseases have occurred in China. The African swine fever (ASF) outbreak in 2018, in particular, exhibited high virulence and strong environmental resistance. It spread through multiple pathways, and no effective vaccine was available for prevention. Characterized by high mortality and high infectivity, the disease not only caused significant losses for many farmers but also altered the industrial structure and market dynamics of China’s hog farming sector, leading to severe impacts on the industry (1). Statistical data indicate that by the end of August 2019, 147 outbreaks of ASF had been reported across all 31 provincial-level regions in China^1^. And approximately 1.2 million pigs were culled by the end of that year. This led to a 27.5% year-on-year decrease in the national live hog inventory compared to 2018^2^, and the hog industry has witnessed an unprecedented fluctuation in production capacity. By the end of 2023, the top 20 large-scale swine enterprises in the country accounted for 27.4% of the total hog output, representing a 16.7 percentage point increase from 2019^3^, which reflects further capitalization in the hog industry. In Das Kapital, Marx proposed that the process of transforming surplus value into capital is termed capital accumulation (2). Capitalization, as an extension and deepening of capital accumulation, refers to the process of converting various equities and assets into capital. Building upon this concept, hog farming capitalization denotes the transformation of inputs or resources in hog production into long-term assets or capital, with its most direct manifestation being the scaling-up of hog farming operations. In pursuit of surplus value, capital-intensive hog farms increase capital input to replace traditional labor factors of production. This essentially reflects the practical application of Marx’s theory of the organic composition of capital in the hog industry. Particularly since the outbreak of ASF, a large number of small-scale farms withdrew from the market due to unprofitability (3). In contrast, larger capitalized farms, benefiting from greater financial resources, technological capabilities, and managerial advantages (4), exhibited higher resilience to the epidemic’s impacts. The proportion of market hogs supplied by farms with an annual output of fewer than 500 head dropped sharply from 57% in 2017 to 35% in 2022^4^. This shift represented a production capacity transfer of approximately 150 million head. The exit rate of small-scale farmers averaged about 7.4 percentage points per year, far exceeding the natural attrition rate. Consequently, the ASF outbreak has objectively accelerated the capitalization process in hog farming. Against this backdrop, examining the accelerating effects and underlying mechanisms of ASF on hog farming capitalization holds substantial practical significance.

Existing studies have extensively explored the impacts of ASF outbreaks and the capitalization of hog farming. Regarding the effects of ASF epidemics, scholars have found that the containment measures implemented following ASF outbreaks may compel farmers to exit the market, leading to reduced pork supply (5, 6). Concurrently, restrictions on cross-regional transportation of live pigs imposed after the outbreaks have resulted in supply shortages, causing significant price fluctuations in the pork market (7, 8). Some scholars have also pointed out that the shrinking demand for pork, coupled with the disruption of hog farming in epidemic areas, will lead to a decrease in resource allocation efficiency (9). In the short term, this further results in a reduction in pork supply. Regarding the capitalization of hog farming, existing studies suggest that large-scale capital-intensive farming can mitigate fluctuations in livestock output and prices (10). Specifically, the entry of industrial capital has prolonged the hog price cycle, contributing to a more stable and sustainable development of the hog industry. In the process of hog farming capitalization, labor opportunity costs serve as a key driving factor (11, 12), while high land costs represent a major constraint (3). Furthermore, some qualitative studies indicate that economies of scale and technological advances are key drivers of capital expansion in livestock farming, while farm consolidation and land transfer are constraints to the scaling of hog farms (13, 14). Regarding the mechanism by which ASF influences hog farming capitalization, current research primarily focuses on two aspects. First, the severe shock of ASF outbreaks has forced numerous small-scale farmers to exit the market (15). This is largely because smallholders typically operate with weaker biosecurity measures and limited disease prevention capabilities. Second, large-scale capital-intensive farms, benefiting from strong financial backing and advanced disease control systems, have rapidly dominated the market post-outbreak (16). Moreover, due to their substantial prior investments in fixed assets, these farms are less likely to adopt abrupt exit strategies. In summary, research on the impact of ASF on the capitalization of hog farming primarily focuses on two aspects: the severe effects on small-scale farmers and the comparative advantages of large-scale capitalized enterprises.

Existing studies have extensively examined the effects of ASF on hog market prices, production volatility, and industry development. However, there is relatively little research on the impact of ASF on the capitalization of hog farming. Given the pronounced acceleration in farming capitalization following the 2018 ASF outbreak, this study investigates the mechanisms and pathways through which ASF influences hog farming capitalization, offering a novel perspective on the determinants of agricultural capital transformation. This study makes the following three main marginal contributions: First, in terms of the research subject, this study focuses on ASF, and combines Marx’s theory of capital composition to clarify the meaning of capitalization. It also explores the mechanism by which ASF affects the capitalization of hog farming, thereby enriching the related research. Second, regarding research content, the paper investigates the impact of ASF on hog farming capitalization from the perspectives of industrial agglomeration and government support, using a moderation effect model to expand the understanding of its pathways. Third, methodologically, this paper employs provincial panel data from 2013 to 2023 and applies multiple empirical approaches, including fixed-effects models, moderation effect models, and difference-in-differences analysis, to provide a more detailed logical framework for understanding the relationship between ASF and hog farming capitalization.

## 2 Theoretical analysis and hypothesis

### 2.1 The accelerating effect of African swine fever on the capitalization of hog farming

As a major public health emergency, the ASF outbreak has exerted significantly more severe production capacity reduction, industry disruption, and government response intensity compared to other swine epidemics (17, 18), presenting unprecedented challenges to China’s hog industry. Paradoxically, this crisis has simultaneously created a unique opportunity to accelerate the capitalization process in China’s hog farming sector.

Firstly, the supply shortage induced by the ASF outbreak triggered an increase in the equilibrium price of the hog market. ASF caused varying degrees of pig mortality or culling across regions, resulting in a precipitous decline in the national hog inventory (19). The information from the Ministry of Agriculture and Rural Affairs indicates that in 2018, there were 99 outbreaks of ASF, resulting in the culling of 800,000 hogs; in 2019, there were 63 outbreaks of ASF, leading to the culling of 390,000 hogs^5^. According to market supply–demand theory, reduced commodity supply leads to an elevated market equilibrium price and decreased equilibrium quantity. Furthermore, since the supply elasticity of hogs is significantly greater than the demand elasticity, when the total supply in the market decreases and consumer preferences remain largely stable, the pig market will experience a situation of insufficient supply and rising prices (20). For instance, in 2019, the price of hogs soared from 10 yuan per kilogram at the beginning of the year to 40 yuan per kilogram by the end of the year, reaching an all-time high (see text footnote 2). Consequently, under the precondition of stable demand and reduced supply, the equilibrium price in the live pig market inevitably began to rise, demonstrating the emerging impacts of supply–demand imbalance.

Secondly, the rising hog prices have triggered herd behavior among farmers, characterized by inventory hoarding and reluctance to sell, which has significantly amplified the abrupt surge in the hog market prices. According to price expectation theory, producers adjust current production decisions based on anticipated future market prices (21). As hog prices increased, most farmers collectively delayed slaughtering to capture excess profits. This widespread practice of holding back hogs or secondary fattening led to the emergence of so-called “super-heavy hogs” in the market, with profits per head soaring to 3,000–4,000 yuan^6^, transforming the industry into a highly lucrative sector. The underlying mechanism can be explained through microeconomic principles. According to existing research, the growth of hogs follows the law of diminishing marginal utility. That is, when reaching a certain period of breeding, as the breeding time extends, the marginal weight gain of hogs shows a decreasing trend.

According to the profit objective function in economics, the profit obtained by the breeder can be expressed as:


(1) $$\pi =P\cdot (G_{0}+\int_{h_{0}}^{h}k\frac{1}{e^{h/h_{0}-1}}\mathrm{dh})-\mathrm{FC}-\mathrm{VC}\cdot h$$

In Equation 1, *π* denotes the profit per hog, *P* represents the expected market price of hogs anticipated by farmers after the outbreak of ASF, and $G_{0}$ stands for the market weight of hogs calculated by farmers under the profit maximization objective without the impact of the epidemic. The variable *h* denotes time, where $h_{0}$ refers to the optimal production duration required for profit maximization under normal conditions. The parameter *k* indicates the standard daily weight gain of hogs, while $k\frac{1}{e^{h/h_{0}-1}}$ represents the marginal weight gain as a function of extended production time. *FC* denotes the fixed cost per hog during the production cycle, and *VC* refers to the daily variable cost per hog.

Taking the partial derivative of Equation 1 with respect to *h* gives:


(2) $$\mathrm{d\pi}=P\cdot k\frac{1}{e^{h/h_{0}-1}}-\mathrm{VC}$$

In Equation 2, when dπ = 0, the objective function reaches its maximum value.


(3) $$P\cdot k\frac{1}{e^{h/h_{0}-1}}=\mathrm{VC}$$

From Equation 3, it can be seen that when the *VC* remains constant, if the price of live pigs continues to rise, farmers will choose to extend the breeding period in order to achieve the goal of maximizing profits. The higher the expected price, the longer the delay in selling the hogs.


(4) $$\frac{h_{1}}{h_{0}}=\ln\frac{P\cdot k}{\mathrm{VC}}+1$$

Equation 4 represents the coefficient of variation for the extended production period derived from Equation 3. Here, $h_{1}$ denotes the optimal production duration under disease-induced market conditions, where producers delay marketing in anticipation of rising hog prices. Crucially, higher expected future prices lead to prolonged feeding periods. Consequently, this reduces the number of hogs reaching market weight, thereby decreasing short-term market supply.

Affected by the herd behavior of farmers when selling their livestock, the supply objective function of the entire hog market is:


(5) $$S_{q}=\frac{h_{0}}{h_{1}}\cdot S_{0}$$

In Equation 5, $S_{q}$ denotes the market supply of hogs, while $S_{0}$ represents the baseline market supply prior to the ASF outbreak. Combining Equations 4, 5 yields the hog supply deficit function:


(6) $${\mathrm{\Delta S}}_{q}=S_{0}-S_{q}=S_{0}(1-\frac{1}{1+\ln(P\cdot k/\mathrm{VC})})$$

In Equation 6, ${\mathrm{\Delta S}}_{q}$ represents the supply–demand gap caused by reduced hog slaughter. The higher the farmers’ expected hog price, the greater this supply shortage becomes. On the demand side, as consumers’ pork consumption gradually recovers, the imbalance between supply and demand continues to widen. Consequently, the short-term equilibrium price of hogs experiences a substantial increase, transforming the hog farming industry into an exceptionally profitable sector with unprecedented profit margins.

Finally, the prospect of high returns has encouraged many hog farmers to increase investment and expand herds. Farmers generally tend to focus more on short-term interests in their management, and their responses to market changes are often rather uniform. This herd behavior leads to a situation where, when the profits in the hog farming industry rose, a large number of farmers began to increase their investment, expand their breeding areas, increase their investment in production materials, and accelerate capital accumulation, thereby further accelerating the capitalization process. For instance, the leading hog farming enterprise, Muyuan Group, achieved a net profit of 13.266 billion yuan in 2022 and made an additional investment of 3 billion yuan^7^.

Based on the Cobb–Douglas production function, the new production capacity and profit function in the hog farming industry can be expressed as follows:


(7) $${\mathrm{\Delta Q}}_{h}=AK^{\alpha }L^{\beta }Z^{\gamma }$$

(8) $$\Delta \pi =PAK^{\alpha }L^{\beta }Z^{\gamma }-\mathrm{kK}-\mathrm{\omega L}-\mathrm{\tau Z}$$

In Equations 7, 8, ${\mathrm{\Delta Q}}_{h}$ denotes the additional production capacity, while Δπ represents the incremental profit. Here, *A*, *K*, *L*, and *Z* correspond to technology, capital, labor, and land inputs, respectively, whereas K 、 ω and τ denote the prices of capital, labor, and land. Under a given technological level and optimal production scale, hog producers operate under constant returns to scale. Under these conditions, the inputs of capital, labor and land maintain a fixed proportional relationship, expressed as L=ψK and Z=φK , with the constraint α+β+γ=1 . Equation 9 presents the functional relationship between incremental profit and newly invested capital.


(9) $$\Delta \pi =(\mathrm{PA}\psi^{\beta }\varphi^{\gamma }-k-\omega \psi -\tau \varphi )K$$

**Figure 1:**
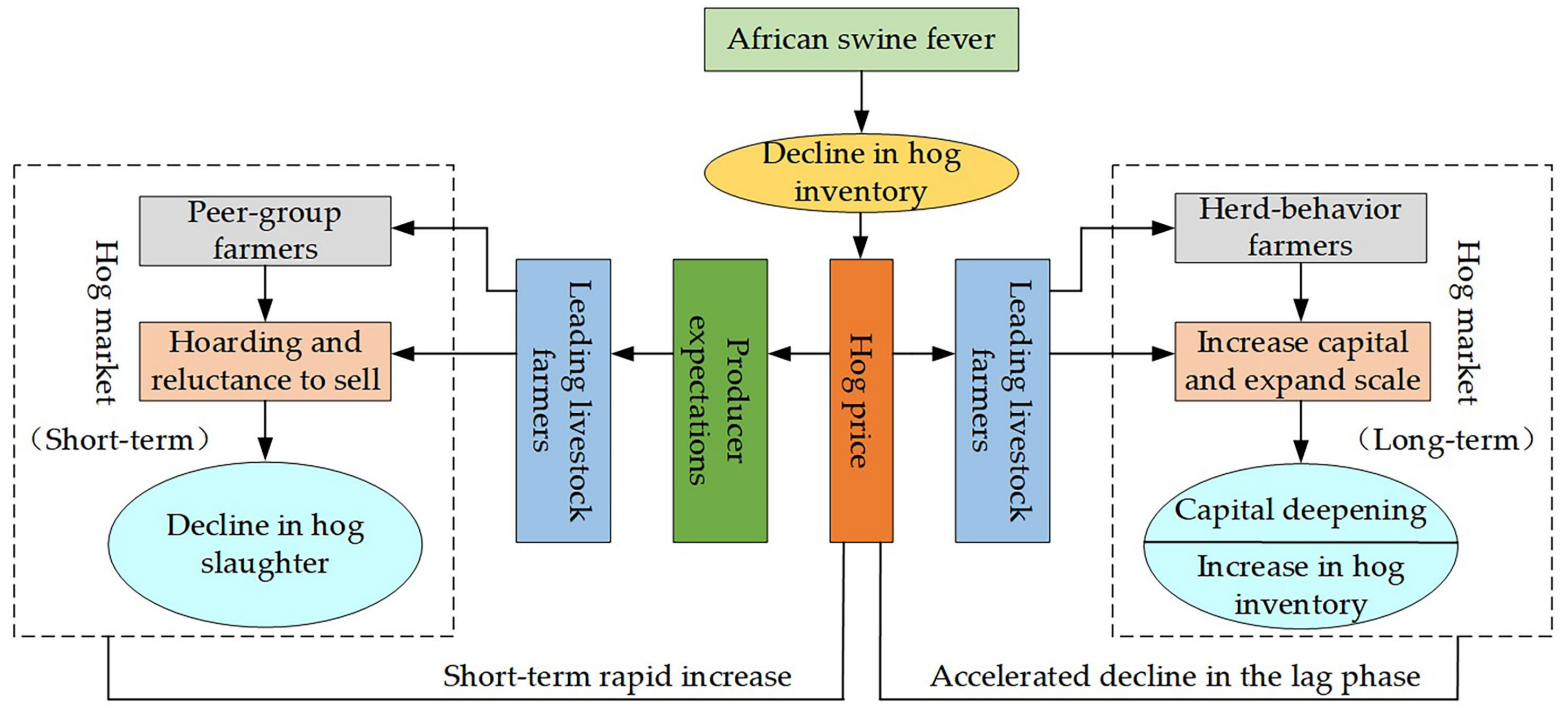
The theoretical logic of how African swine fever outbreaks accelerate the capitalization of hog farming.

Equation 9 demonstrates that increased capital investment leads to higher profits for livestock enterprises. This finding indicates that farmers tend to make additional investments to pursue greater profits, resulting in a significant rise in the capital-to-asset ratio. Consequently, capital exerts stronger substitution effects on labor inputs, further intensifying the capitalization process in the entire industry. In the first three quarters of 2021, China’s hog farming industry reached a market size of 1.21 trillion yuan^8^. Many companies have increased investments not only in farming operations but also expanded into upstream sectors such as feed processing and breeding stock production, as well as downstream sectors including slaughtering and processing. This expansion has driven the integration and development of the entire industry chain. Figure 1 illustrates the theoretical logic of how ASF outbreaks accelerate the capitalization of hog farming.

Based on this, this article proposes Hypothesis H1:

*Hypothesis H1:* The outbreak of ASF can effectively promote the capitalization level of hog farming.

### 2.2 The regulating effect of industrial agglomeration

According to (35) research on The Competitive Advantage of Nations, industrial clustering involves the concentration of interconnected businesses within a specific geographic area, centered around a particular industry. Such clustering generates multiple economic benefits for the hog production industry. A case in point is the Modern Agricultural Industrial Park in Neixiang County, Henan Province. Following the ASF outbreak, leading hog producers in the park, particularly Muyuan Group, experienced rapid expansion. Muyuan’ s annual slaughter volume surged from 11.01 million heads in 2018 to 63.82 million heads in 2023, capturing 8.8% of the national market share^9^.

On the one hand, the geographic concentration of swine enterprises accelerates local infrastructure development. Enhanced infrastructure, in turn, attracts higher-quality enterprises to the cluster, creating a virtuous cycle that strengthens regional industrial competitiveness (11, 12). This agglomeration enables shared utilization of public infrastructure, effectively reducing average production costs through external economies of scale, thereby mitigating the negative impacts of ASF. Furthermore, economically robust hog producers within these clusters have formed strategic alliances through agglomeration effects, facilitating shared access to superior genetic resources and centralized waste treatment facilities (22). The rational allocation of supporting resources has further enhanced the production efficiency of hog farming, prompting enterprises to make additional investments and accelerating the capacity recovery of hog farming enterprises after the outbreak of ASF. On the other hand, capital, technology, and human resources constitute fundamental prerequisites for the development of the hog industry (23). The hog industry agglomeration zone has created favorable conditions for attracting capital, technology, and talent through well-developed infrastructure and government incentives. Furthermore, the industrial agglomeration zone brings together various market entities, including government agencies, hog farming enterprises, and research institutions. Through mutual collaboration and resource sharing, these entities generate synergistic innovation, facilitating the creation, dissemination, and application of innovative research outcomes such as advanced hog farming techniques and pollution treatment technologies. This collective effort enhances their capacity to jointly mitigate the impact of epidemics. This not only provides more impetus and favorable conditions for hog farming enterprises to scale up and attract capital, but also draws in a group of highly skilled professionals.

In conclusion, after the outbreak of ASF, hog farming enterprises in regions with a high degree of industrial concentration will accelerate their capitalization process due to the advantages brought by their location in the concentrated area.

Based on this, this article proposes Hypothesis H2:

*Hypothesis H2:* As the degree of industrial agglomeration increases, the promoting effect of ASF on the capitalization of hog farming will also strengthen.

### 2.3 The moderating effect of government support

The government plays a strategic guiding and macro-steering role in the market economy. By formulating and implementing specific incentive policies and measures, as well as promoting entrepreneurship and advocating for corporate social responsibility, it influences the behavior of individuals and enterprises. These actions are designed to drive regional industrial development, enhance market efficiency, and maximize social welfare (24). Consequently, the capitalization process of the regional hog farming industry is inevitably shaped by government interventions.

First, to mitigate the impact of the pandemic, local governments implemented a series of subsidy and support policies, providing a stable external environment for the steady and healthy development of the hog farming industry (25, 26). These policies also offered financial assistance to swine enterprises, facilitating rapid production recovery. For instance, the government encouraged financial institutions to provide loan support to the hog farming sector, including credit assistance for large-scale farms and policies ensuring continued lending and non-withdrawal of credit for temporarily distressed enterprises. Such measures helped hog farming firms cope with the negative shocks caused by ASF. Moreover, increased fiscal support from the government strengthened the capital capacity of enterprises, promoting production recovery and enhancing the scale of intensive farming operations.

Second, the government has strengthened the development of a socialized service system for hog farms, promoting the expansion, quality improvement, and efficiency enhancement of such services in the industry (27). Multi-faceted and multi-tiered services, including technical guidance, market intelligence, quality monitoring, and disease prevention and control, not only enhance production efficiency and product quality for hog farming enterprises but also enable timely responses to market fluctuations and disease risks, thereby mitigating potential losses. Following the ASF outbreak, the government provided guidance-oriented services, assisting enterprises in adjusting production plans. These measures contributed to containing large-scale disease transmission and maintaining stable industry operations.

Finally, to support the development of the livestock breeding industry, the government has provided land support policies for qualified hog farms and strengthened the support for farmers. In 2019, the Ministry of Natural Resources issued the “Notice on Ensuring Land for hog Breeding,” clearly stating that hog breeding land can use general farmland, and as long as it is used for breeding purposes, there is no need for land occupation and compensation balance. Moreover, the scale of ancillary facilities land has been increased, and the upper limit of 15 acres has been abolished^10^. This not only boosts the production enthusiasm of farmers, promotes the development of hog breeding towards large-scale and intensive models, but also provides a guarantee for the stable supply of pork to the market and people’ s livelihood needs. At the same time, the stable policy environment also helps swine enterprises to make long-term plans and investments, and attracts long-term capital.

Based on this, this article proposes Hypothesis H3:

*Hypothesis H3:* Government support exerts a positive moderating effect on the role of ASF in promoting the capitalization of hog farming.

## 3 Methods and data

### 3.1 Date sources

The study utilizes panel data from 30 Chinese provinces (excluding Hong Kong, Macao, Taiwan, and Tibet; data for Xinjiang Uygur Autonomous Region includes Xinjiang Production and Construction Corps) spanning 2013 to 2023. Provincial year-end live hog inventory data were obtained from the China Statistical Yearbook. Metrics for the organic composition of capital in hog farming operations were sourced from the National Agricultural Product Cost–Benefit Compilation. Control variables including urbanization rate, transportation accessibility, per capita pork consumption, and labor opportunity costs were extracted from the China Statistical Yearbook, while cultivated land carrying capacity indicators is derived from China Agricultural Yearbook. In the measurement of the regulating variables, the data of total output value of agriculture, forestry, animal husbandry and fishery, as well as total output value of hog farming, are derived from China Rural Statistical Yearbook, while the data of gross national product and fiscal expenditure are obtained from China Statistical Yearbook. Missing data points were addressed using interpolation methods.

### 3.2 Model setting

#### 3.2.1 Benchmark regression model

To investigate the impact of ASF on the capitalization of hog farming, the following baseline regression model was constructed:


(10) $$\ln Y_{\mathrm{it}}=\alpha_{0}+\alpha_{1}(S_{i}\times Q_{t})+\alpha_{2}\text{Control}s_{\mathrm{it}}+\delta_{i}+\mu_{t}+\varepsilon_{\mathrm{it}}$$

In Equation 10, *i* and *t* denote region and year, respectively, where $\ln Y_{\mathrm{it}}$ represents the natural logarithm of the degree of capitalization in hog farming. The interaction term $S_{i}\times Q_{t}$ serves as the core explanatory variable in this study. Here, $S_{i}$ indicates the reduction rate in year end hog inventories, with $S_{i}=1$ assigned to the treatment group (regions exhibiting *a* ≥ 25% reduction in annual pig slaughter) and $S_{i}=0$ to the control group (regions with <25% reduction). $Q_{t}$ is a time dummy variable for the ASF outbreak period, where $Q_{t}=1$ for *t* ≥ 2018 and 0 otherwise. The vector $\text{Control}s_{\mathrm{it}}$ encompasses a set of control variables affecting hog farming capitalization, while $\delta_{i}$ and $\mu_{t}$ represent region-fixed effects and year-fixed effects, respectively. $\varepsilon_{\mathrm{it}}$ denotes the error term. The coefficients $\alpha_{0}$ , $\alpha_{1}$ , and $\alpha_{2}$ are parameters to be estimated, with $\alpha_{1}$ being the focal parameter that captures the net effect of ASF on hog farming capitalization after controlling for confounding factors.

#### 3.2.2 Regulatory effect model

Based on Equation 10, we incorporate government support and industrial agglomeration as moderating variables, along with their interaction terms with ASF, to test their positive regulatory effects on capitalization of hog farming. The specific regression model is as follows:


(11) $$\begin{array}{l} \ln Y_{\mathrm{it}}=\beta_{0}+\beta_{1}(S_{i}\times Q_{t})+\varphi K_{\mathrm{it}}+\eta (S_{i}\times Q_{t}\times K_{\mathrm{it}})+\\ \;\;\;\;\;\;\;\;\;\;\;\;\;\;\;\;\;\beta_{2}\text{Control}s_{\mathrm{it}}+\delta_{i}+\mu_{t}+\varepsilon_{\mathrm{it}} \end{array}$$

In Equation 11, $K_{\mathrm{it}}$ denotes the moderating variables, including industrial agglomeration and government support, with φ representing their corresponding coefficients. The interaction term $S_{i}\times Q_{t}\times K_{\mathrm{it}}$ captures the moderating effects between the core explanatory variable and these moderators, where η measures their joint influence. The coefficients $\beta_{0}$ and $\beta_{1}$ are parameters to be estimated, while all other variables and parameters remain consistent with those defined in Equation 10.

### 3.3 Variable selection

#### 3.3.1 Explained variable: capitalization of hog farming

According to Marx’s theory of the organic composition of capital, the organic composition of capital refers to the value composition of capital determined by its technical composition, which reflects changes in technical conditions. It is typically expressed by the ratio *C*:V, where *C* represents constant capital (the value of means of production) and *V* denotes variable capital (the value of labor power). A higher organic composition of capital indicates greater means of production employed per unit of labor, reflecting a more capital-intensive production process and a higher degree of capitalization. Based on this theoretical framework, this study adopts the organic composition of capital in hog farms as a metric to evaluate the capitalization level of hog production, with the specific measurement approach detailed as follows:


(12) $$Y_{\mathrm{it}}=C_{\mathrm{it}}/V_{\mathrm{it}}$$

In Equation 12, $Y_{\mathrm{it}}$ represents the organic composition of capital in hog farms, which serves as the proxy for the level of capitalization in hog production in this study. Here, $C_{\mathrm{it}}$ denotes constant capital, measured by the depreciation of fixed assets per hog, while $V_{\mathrm{it}}$ indicates variable capital, represented by the labor cost per hog. As the capital accumulation of farmers expands, capitalization of hog farming can be observed in all scales. However, due to the severe lack of data for many small-scale farmers, this article selects the capitalization level of hog farming in scaled farms as the dependent variable of the article. According to the classification by the Ministry of Agriculture and Rural Affairs, hog farms with an annual slaughter capacity of over 500 head are classified as scaled farms. Therefore, the depreciation of fixed assets and labor costs in the text represent average values for both large-scale and medium-scale farms. Large-scale pig farms are defined as those with an annual slaughter capacity exceeding 1,000 head, while medium-scale farms have an annual slaughter capacity between 100 and 1,000 head.

#### 3.3.2 Explained variable: African swine fever

This study employs the year 2018, marking the outbreak of ASF, as the threshold for the time dummy variable. The core explanatory variable is represented by the interaction term $S_{i}\times Q_{t}$ , where $S_{i}$ denotes the dummy variable for regions affected by ASF and $Q_{t}$ indicates the dummy variable for the time point of the outbreak of the epidemic. Specifically, the interaction term equals 1 if a province is both classified as a region affected by ASF and the observation period falls after the outbreak, and 0 otherwise. To operationalize the ASF impact variable, this study follows the methodology of Hao et al. (28), defining treatment and control groups based on the decline rate in year-end hog inventories: provinces experiencing a ≥ 25% reduction are assigned to the treatment group, while the remaining provinces serve as the control group. The reason for choosing this classification criterion is that the reduction in hog inventory in the control group provinces was less than one quarter, indicating that they were relatively less affected by the ASF, which meets the classification requirements for the experimental group and the control group as stipulated by the difference-in-differences method. Moreover, the 25% threshold ensures adequate sample sizes for both treatment and control groups.

#### 3.3.3 Regulating variable: government support and industrial agglomeration

To measure industrial agglomeration in the hog farming sector, this study adopts the location quotient index, a method that not only offers operational simplicity but also eliminates scale-related biases by normalizing regional differences. Following the approach of Wu et al. (29), we calculate the location quotient index to quantify regional agglomeration levels of hog farming, as expressed by the following formula:


(13) $$M_{\mathrm{it}}=\frac{e_{\mathrm{it}}/e_{t}}{E_{\mathrm{it}}/E_{t}}$$

In Equation 13, M denotes the industrial agglomeration level, where *e* represents the gross output value of the hog farming sector, and *E* corresponds to the gross domestic product of the region. The subscripts *i* and *t* indicate province and year, respectively.

This study measures government support intensity by the proportion of fiscal expenditure allocated to the hog farming sector, following the methodology of Li (30). The specific measurement procedure involves two steps: First, by multiplying the proportion of the total output value of the regional hog farming industry in the total output value of agriculture, forestry and animal husbandry and fishery by the fiscal expenditure on agricultural, forestry and water affairs in that region, the government’s fiscal expenditure in the hog farming industry can be estimated. Second, the intensity of government support is quantified as the ratio of hog farming fiscal expenditure to the total general budgetary expenditure of local governments.

#### 3.3.4 Control variable

To account for potential confounding factors affecting hog farming capitalization, this study incorporates the following control variables based on established literature: ① Urbanization rate: Reflects regional development stages; ② Transport accessibility: Measures regional investment attractiveness; ③ Arable land carrying capacity: Indicates potential for livestock development; ④ Per capita pork consumption: Proxies for local living standards and dietary patterns; ⑤ Labor opportunity cost: Captures regional non-agricultural economic development. The specific measurement methods and descriptive statistics of each variable are shown in Table 1.

**Table 1:** Variable definitions and descriptive statistics results.

Variable name	Variable measurement	Mean	Min	Max
Capitalization of hog farming	The ratio of fixed asset depreciation to labor cost required for large and medium-sized hog farms	0.155	0.023	0.713
Industrial agglomeration	Location entropy index measurement	1.098	0.010	3.669
Government support	The financial support provided by the regional government for hog farming	0.011	0.001	0.029
Urbanization rate	Ratio of urban population to total population	0.619	0.379	0.896
Transport accessibility	The mileage of railways, highways and inland waterways in each province and the ratio of these distances to the area of each province	1.060	0.101	2.513
Arable land carrying capacity	The ratio of the cultivated land area of each province to the total cultivated land area of the country	0.032	0.001	0.135
Per capita pork consumption	Per capita pork consumption/kilogram	20.596	3.132	48.520
Labor opportunity cost	Per capita wage income of rural residents / 10,000 yuan	0.692	0.131	2.702

## 4 Results

### 4.1 Baseline regression analysis

Using Stata 17.0, we estimated regression model (10) to examine the impact of ASF outbreaks on the capitalization process in hog farming. The regression results are presented in Table 2. Column (1) reports the baseline regression without control variables, controlling only for region and year fixed effects. The coefficient of the core explanatory variable is positive and statistically significant at the 1% level, indicating that ASF significantly accelerates the capitalization of hog farming. In Column (2), we further incorporate control variables, including urbanization rate, transportation accessibility, arable land carrying capacity, per capita pork consumption, and labor opportunity costs. The results demonstrate that the ASF variable remains positive and significant at the 1% level, suggesting that even after controlling for these factors, ASF outbreaks continue to exhibit a significant promoting effect on hog farming capitalization. These findings support Hypothesis H1. From the regression results, it can be observed that the high profits brought about by the shortage of supply in the hog market have attracted a large amount of social capital to flow into the hog farming industry. At the same time, the technical threshold of the hog breeding industry has further increased with the outbreak of the epidemic, and traditional hog farmers who cannot adapt to modern and industrialized breeding models have been eliminated from the market first. This has further accelerated the capitalization process of the originally stable hog industry after the outbreak of ASF. As shown in column (2), the urbanization rate, transportation convenience and labor opportunity cost have a significant positive impact. The reason may be that urbanization has expanded the market for hog farming, attracting more capital investment into the sector. This has promoted a shift from low-efficiency backyard farming to a capital-intensive model characterized by high investment and advanced technology. Meanwhile, convenient transportation can reduce logistics time and costs, helping to expand the sales radius of the hog market and increase profit margins. Therefore, areas with convenient transportation are more likely to attract external investment and accelerate the capitalization process of hog breeding. At the same time, when the labor opportunity cost rises, enterprises are more inclined to use automated equipment to replace manual labor to achieve large-scale production and reduce labor costs. The capitalization of hog breeding is an inevitable result.

**Table 2:** Baseline regression result.

Variable	Capitalization of hog farming			
	(1)		(2)	
	Coefficient	Robust standard error	Coefficient	Robust standard error
Interaction term	0.146***	0.052	0.175***	0.054
Urbanization rate	—	—	1.133**	0.511
Transport accessibility	—	—	0.609**	0.272
Arable land carrying capacity	—	—	−0.038	0.127
Per capita pork consumption	—	—	0.189	0.142
Labor opportunity cost	—	—	0.632***	0.185
Constant term	−2.222***	0.043	−2.646***	0.679
Region control	Yes		Yes	
Year control	Yes		Yes	
R^2^	0.157		0.214	
Observations	330		330	

***, **, and * represent significance tests at the 1, 5, and 10% levels, respectively.

### 4.2 Parallel trend test

**Figure 2:**
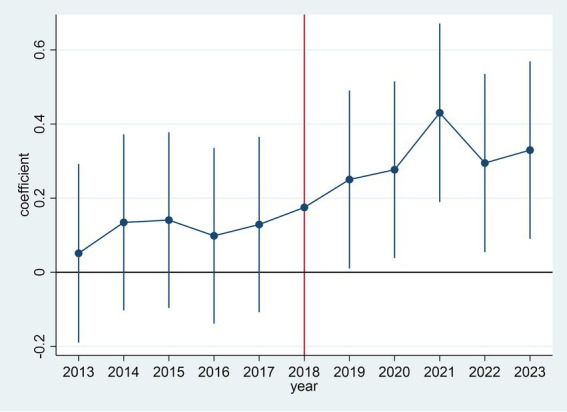
Parallel trend test result.

The parallel trend assumption posits that, in the absence of intervention, the treatment group would have followed a similar trend as the control group. Following the methodology of Lu et al. (31), we conducted parallel trend tests to examine the dynamic effects of ASF outbreaks on hog farming capitalization. Figure 2 shows parallel trend test result. As illustrated in Figure 2, the regression coefficients of interaction terms for the five pre-treatment periods and the outbreak period itself are statistically indistinguishable from zero. However, these coefficients become significantly positive in the post-outbreak periods. This indicates that before the outbreak of ASF, there was no significant difference in the process of piglet breeding capitalization between the experimental provinces and the control provinces. This satisfies the parallel trends assumption, meeting the prerequisite for employing a difference-in-differences analysis.

### 4.3 Robustness tests

#### 4.3.1 Replace the explained variable

To verify the robustness of the research conclusions, this study conducts robustness tests by replacing the explained variable. Generally speaking, large-scale hog farms exhibit higher capital intensity; thus, the number of such farms can, to some extent, reflect the capitalization status of the hog industry. In accordance with the National Modern Agricultural Development Plan (2011–2015), which uses the proportion of farms with an annual output of 500 heads or more relative to the total number of farms as a measure of farming scale, this paper defines farms with an annual output of 500 heads or more as capitalized farming operations. Therefore, the explained variable is replaced with the proportion of capitalized farms, specifically the proportion of hog farms with an annual output of 500 heads or more. Consequently, the capitalization rate of hog farming is calculated as follows:


(14) $$L=\sum_{n=3}^{6}S_{n}/\sum_{n=1}^{6}S_{n}$$

In Equation 14, *L* represents the capitalization rate of hog farming. According to the China Animal Husbandry and Veterinary Yearbook, large-scale hog farming is classified into six groups, where $S_{n}$ denotes the number of farms in the nth group. Column (1) in Table 3 presents the regression results after replacing the explained variable. The estimated coefficient of ASF on the proportion of capitalized farms is significantly positive, indicating that ASF significantly accelerates the capitalization process of hog farming. This finding supports the conclusions derived from the baseline regression model.

**Table 3:** Robust test results.

Variable	Capitalization of hog farming					
	(1)	(2)	(3)	(4)	(5)	(6)
	Replace the explained variable	Proximity matching	Nuclear matching	Adjust the threshold for classification	First stage	Second stage
IV					0.794*** (0.016)	
Interaction term	0.042** (0.018)	0.387*** (0.116)	0.275*** (0.070)	0.171*** (0.055)		0.242* (0.139)
Constant term	−0.687*** (0.231)	−5.293** (2.143)	−4.316*** (1.273)	−2.751*** (0.676)		
Control variables	Yes	Yes	Yes	Yes	Yes	Yes
Region control	Yes	Yes	Yes	Yes	Yes	Yes
Year control	Yes	Yes	Yes	Yes	Yes	Yes
F test					363.49 (0.000)	
Cragg-Donald Wald F statistic						431.453 [16.38]
Kleibergen Paap rk LM statistic						20.096 (0.000)
R^2^	0.377	0.363	0.247	0.212		0.925
Observations	330	147	228	330	330	330

***, **, and * represent significance tests at the 1, 5, and 10% levels, respectively. The values in parentheses are the robust standard errors. The values in square brackets are the critical values at the 10% significance level. For the F test and the test for unidentifiable instrumental variables, the values in the small brackets are the p-values.

#### 4.3.2 Placebo test

**Figure 3:**
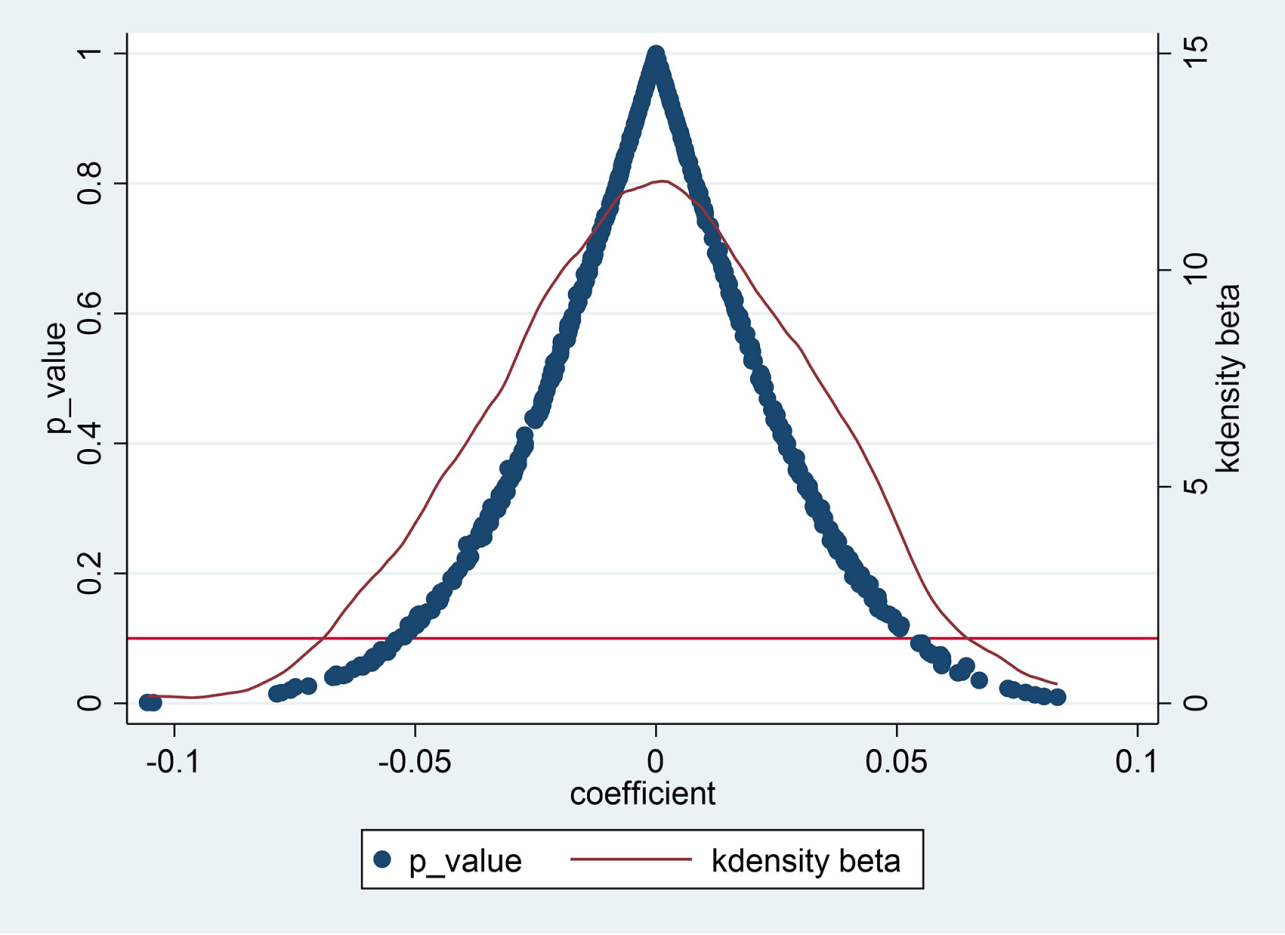
Placebo test result.

To eliminate the interference caused by omitted variables and unobserved factors, and to ensure that the conclusion drawn in the previous part is due to the outbreak of ASF, this study conducts a placebo test. The underlying rationale involves randomizing the treatment and control groups and then re-estimating the baseline regression. If the key coefficient remains statistically insignificant across numerous randomizations, it suggests that the observed effect of the core explanatory variable is not due to random chance. This study followed the approach of Lu et al. (32), constructing interaction terms in the sample provinces and conducting 500 sampling tests to perform a placebo test. The placebo test results are shown in Figure 3. It can be seen from this that after 500 sampling regressions, the estimated coefficients of the core explanatory variables follow a normal distribution, and most of the *p*-values are greater than 0.1. This indicates that randomizing the occurrence of ASF yields no statistically significant effect, confirming that the positive impact of ASF on hog farming capitalization is robust and not attributable to spurious correlations. Thus, the placebo test validates our baseline findings.

#### 4.3.3 Propensity score matching test

Although the difference-in-differences approach effectively captures the relative changes in hog farming capitalization before and after the ASF outbreak, and controlling for regional and year-fixed effects mitigates some endogeneity concerns, potential biases arising from “selection bias” remain unresolved. Therefore, in order to ensure the robustness of the research conclusions, the article further employs the propensity score matching method to correct the endogeneity issues caused by sample selection bias. In Table 3, columns (2) and (3) respectively represent the results after 1:3 neighboring matching and kernel matching. It can be seen that after controlling for sample selection bias, the core explanatory variable remains significantly positive. This robustness check further validates the reliability of our baseline regression results.

#### 4.3.4 Adjust the threshold for dividing the experimental group from the control group

To further ensure the robustness of our findings, this study redefines the classification threshold to distinguish between the treatment and control groups. Provinces are assigned to the treatment group when their year-end live hog inventory shows a decline of 30% or greater, while others constitute the control group. The regression results using this adjusted threshold are presented in Column 4 of Table 3. From the results, it can be seen that by using whether the decrease in the end-of-year hog inventory was greater than or equal to 30% as the classification criterion to distinguish the experimental group from the control group, the regression coefficient remained significantly positive at the 1% level, thus confirming the robustness of the conclusion of the previous study.

#### 4.3.5 Endogeneity test

To examine potential endogeneity between ASF and the capitalization of hog production, this study adopts the methodology of Li and Li (33), employing a one-period lagged ASF variable as an instrumental variable (IV) and conducting two-stage least squares estimation. The results are presented in Columns (5) and (6) of Table 3. The first-stage regression confirms that the instrumental variable significantly promotes the capitalization of hog production, with statistical significance at the 1% level. Additionally, both the weak instrument test and the underidentification test support the validity of the chosen IV, further substantiating the robustness of our empirical approach. Therefore, after addressing potential endogeneity concerns, we conclude that ASF continues to exhibit a significant positive effect on the capitalization of hog production, reinforcing the reliability of our baseline findings.

### 4.4 Mechanism analysis

To investigate whether the two moderating variables, industrial agglomeration and government support, play a role in the mechanism through which ASF influences the capitalization of hog farming, the study incorporates these moderating variables into Equation 11 for regression analysis. Table 4 presents the results of the moderating effect tests. Column (1) shows that the estimated coefficient of the interaction term between ASF and industrial agglomeration is significantly positive, indicating a positive moderating effect of industrial agglomeration. Specifically, a higher degree of industrial agglomeration strengthens the promoting effect of ASF on hog farming capitalization. This finding suggests that hog farming enterprises in agglomerated regions gain greater developmental advantages. Moreover, the agglomeration of hog farming accelerates the exit and consolidation of small and medium-sized farms, leading to a development pattern characterized by “one dominant player and several strong competitors.” Consequently, more small and medium-sized farmers are forced to exit or merge. Hypothesis H2 is thus validated. Column (2) reveals that after introducing the interaction term between ASF and government support, the regression coefficients of both the core explanatory variable and the interaction term are significantly positive. This robustly demonstrates that government support exerts a positive moderating effect on the accelerating role of ASF in hog farming capitalization. In other words, under the shock of a major animal epidemic, targeted government intervention can effectively stimulate market vitality and drive the industry toward large-scale, capital-intensive transformation. Hypothesis H3 is thereby confirmed.

**Table 4:** Adjustment effect test.

Variable	Capitalization of hog farming			
	(1)		(2)	
	Coefficient	Robust standard error	Coefficient	Robust standard error
Interaction term	0.203***	0.054	1.322***	0.334
Interaction term × Industrial Agglomeration	0.157***	0.040	—	—
Industrial Agglomeration	−0.077	0.078	—	—
Interaction term × Government Support	—	—	0.245***	0.071
Government Support	—	—	0.062	0.081
Constant term	−3.526***	0.731	−3.529***	0.695
Control variables	Yes		Yes	
Region control	Yes		Yes	
Year control	Yes		Yes	
R^2^	0.263		0.268	
Observations	330		330	

***, **, and * represent significance tests at the 1, 5, and 10% levels, respectively.

### 4.5 Heterogeneity tests

#### 4.5.1 Analysis based on the heterogeneity of capital stock

Capital stock serves as a critical indicator for measuring the capitalization of the hog farming industry. To examine whether the impact of ASF on hog farming capitalization varies significantly across regions with different capital stock levels, this study draws on the research methods and parameter settings of Li (34), and uses the perpetual inventory method to calculate the capital stock of the hog farming industry in each region. The sample was then divided into two groups based on the median of the 10-year average capital stock across 30 provinces, followed by group-wise regression analysis. The results, presented in Columns (1) and (2) of Table 5, demonstrate that in regions with higher capital stock, the promoting effect of ASF on hog farming capitalization is statistically significant at the 5% level, whereas no significant effect is observed in regions with lower capital stock. A possible explanation is that regions with greater capital stock typically exhibit higher industrial concentration and possess superior developmental advantages in hog farming, thereby providing a solid foundation for enterprises to accelerate capitalization. In other words, a higher capital stock may amplify the accelerating effect of ASF on hog farming capitalization.

**Table 5:** Heterogeneity tests.

Variable	Capitalization of hog farming			
	(1)	(2)	(3)	(4)
	High capital stock	Low capital stock	Major corn-producing regions	Non-major corn-producing regions
Interaction term	0.140** (0.062)	0.156 (0.097)	0.107 (0.067)	0.378*** (0.097)
Constant term	−0.378 (1.367)	−3.077*** (0.850)	−4.294*** (0.896)	−2.777** (1.137)
Control variables	Yes	Yes	Yes	Yes
Region control	Yes	Yes	Yes	Yes
Year control	Yes	Yes	Yes	Yes
R^2^	0.533	0.197	0.302	0.286
Observations	165	165	165	165

***, **, and * represent significance tests at the 1, 5, and 10% levels, respectively. And the values in parentheses are the robust standard errors.

#### 4.5.2 Analysis based on the heterogeneity of resource endowments

Corn, as the primary feed ingredient in hog farming, is characterized by its high energy content, palatability, and digestibility. Owing to its resource endowment advantages, major corn-producing regions can facilitate the relocation of hog production capacity to these areas, thereby achieving organic integration of corn cultivation and hog farming and enhancing breeding efficiency. Based on the average corn yield from 2013 to 2023 across the sample regions, this study categorizes the 30 selected provinces into major corn-producing regions and non-major corn-producing regions using the median value. The regression results, presented in Columns (3) and (4) of Table 5, indicate that in non-major corn-producing regions, the accelerating effect of ASF on hog farming capitalization is statistically significant at the 1% level, whereas no significant effect is observed in major corn-producing regions. A plausible explanation lies in the fact that major corn-producing regions are often major grain-producing areas and key hog farming provinces, where agricultural and livestock infrastructure is well-developed, policy support is robust, and resource endowments are favorable. Consequently, these regions already exhibit a relatively high degree of hog farming capitalization, leaving limited room for further enhancement due to ASF. In contrast, non-major corn-producing regions lack inherent advantages in hog farming and development, resulting in a lower baseline capitalization level. This disparity grants non-major corn-producing regions a late-mover advantage, making the promoting effect of ASF on hog farming capitalization more pronounced.

## 5 Research conclusions, discussion and policy recommendations

### 5.1 Research conclusions

Based on panel data from 30 provinces in China between 2013 and 2023, this study examines the impact of ASF on the capitalization of hog farming. A fixed-effects model, a moderating effects model, and a difference-in-differences approach are employed for empirical analysis. The main findings are as follows: First, ASF accelerated the capitalization process of hog farming. Provinces severely affected by ASF experienced a faster increase in capitalization compared to less affected provinces. This conclusion remains robust after a series of robustness tests. Second, mechanism tests reveal that the acceleration effect was more pronounced in regions with higher industrial agglomeration, indicating that industrial agglomeration provides advantages for capitalization. Additionally, government support played a positive moderating role in the relationship between ASF and capitalization. Finally, the accelerating effect was stronger in provinces with higher capital stock in hog farming and non-major corn-producing regions. In contrast, the effect was relatively limited in provinces with lower capital stock and major corn-producing regions.

### 5.2 Discuss further

The capitalization of hog farming represents an advanced stage of industrial evolution and serves as a critical driver for high-quality development in the sector. When the capitalization process progresses steadily, it facilitates the realization of economies of scale, enhances production efficiency in the hog industry, and strengthens the sector’s resilience to external risks. However, when the process of capitalization progresses too rapidly, the massive inflow of capital may lead to a rapid expansion of production capacity in the hog farming industry, resulting in an oversupply of hogs in the market and causing a prolonged period of low prices, leading to the emergence of a “super cycle.” Under capital influence, the traditional regularity of hog cycles is gradually diminishing. In scenarios of accelerated capitalization, major animal epidemics act as an “initiator,” herd behavior such as inventory hoarding functions as an “amplifier,” and crowd-following investments in capacity expansion serve as an “accelerator.” Conversely, insufficient capitalization exacerbates instability in production capacity and price volatility in the hog market, hindering technological innovation and competitiveness among swine enterprises. Therefore, to ensure the stable operation of the hog farming industry, it is imperative to advance capitalization steadily under the principle of sustainable development.

### 5.3 Policy suggestion

First, strengthen the epidemic prevention and control system to enhance the emergency response capacity of the hog industry. In the face of the swine epidemic, the central government should strengthen financial and fiscal support, set up special fiscal funds, and guide banks and other financial institutions to increase credit supply to the hog industry, thereby reducing the financing costs for farmers. Local governments and relevant authorities should adopt a strategy of hierarchical classification and precise prevention and control, establish a three-level epidemic prevention grid system at the county, township and village levels, implement “one farm, one file” digital management for hog farms with a breeding capacity of over 500 heads, ensure “early detection, early reporting, early diagnosis, early handling, and early recovery”^11^, reduce the impact of the epidemic on the hog market, and ensure market supply.

Second, prioritize restorative capacity growth while preventing excessive expansion in hog production. On the one hand, to alleviate the supply shortage caused by the herd-like behavior of “hoarding and refusing to sell” in the industrialized areas, as well as the blind expansion of hog production capacity triggered by the conformist behavior of “increasing investment and expanding facilities,” a production capacity early warning mechanism should be established. For the industrialized areas that have caused severe overcapacity, regional restrictions on project approvals should be implemented. When the warning line is triggered, the approval of new projects in the industrialized areas should be suspended. On the other hand, the government should guide rational capacity adjustment and restorative growth in the hog industry. Large-scale hog farming enterprises should be encouraged to optimize production scale and structure, while an excess progressive tax could be levied on overexpanded operations. Additionally, reducing direct subsidies for oversized enterprises would help deter disorderly capacity expansion.

Third, multi-channel collaboration should be enhanced to elevate the capital market participation of the hog industry and promote its high-quality development. This involves strengthening technological coordination and innovation within industrial clusters, improving infrastructure and public service systems, and unifying the provision of epidemic prevention technologies, feed supplies, and sales channels to maximize agglomeration benefits. Such integration will transform the challenges posed by epidemics into core drivers for industrial upgrading. Meanwhile, governments should refine fiscal subsidies with precision, targeting critical sectors while adjusting support levels based on epidemic dynamics. The establishment of a “Special Loan Program for Epidemic Prevention and Industrial Upgrading in the Hog Industry” and the expansion of ASF insurance coverage are recommended^12^. These measures will accelerate the industry’s transition from “passive epidemic response” to “proactive upgrading”.

## Data Availability

Publicly available datasets were analyzed in this study. This data can be found here: BRIK Agricultural Database http://www.agdata.cn. The raw data supporting the conclusions of this article will be made available by the authors, without undue reservation.

## Footnotes

^1^ https://www.sohu.com/a/346858263_223261

^2^ http://www.agdata.cn

^3^ https://www.sohu.com/a/751888908_211780

^4^ www.moa.gov.cn

^5^ http://www.knowcat.cn/p/20241118/1863008.html

^6^ http://www.caaa.cn/

^7^ https://www.sohu.com/a/673702472_120088066

^8^ https://data.eastmoney.com/report/zw_industry.jshtml?infocode=AP202201201541567953

^9^ https://baijiahao.baidu.com/s?id=1804079404325810120&wfr=spider&for=pc

^10^ https://www.gov.cn/zhengce/zhengceku/2019-11/22/content_5454574.htm

^11^ https://www.gov.cn/zhengce/content/2019-09/10/content_5428819.htm

^12^ https://m.12371.gov.cn/content/2025-02/23/content_484970.html

## References

[1] Shi ZZ Hu XD. African swine fever shock: China's hog industry's resilience and its influencing factors. Animals. (2023) 13:2817. doi: 10.3390/ani13182817, PMID: 37760217 PMC10525915

[2] Marx K. Das Kapital: volume one. Beijing: People's Publishing House (2004).

[3] Huang BK Geng XH. Has the increase in labor opportunity costs been the reason for the capitalization development of pig farming? J Agro-For Econ Manag. (2022) 3:331–41. doi: 10.16195/j.cnki.cn36-1328/f.2022.03.35

[4] Zhou K Wang H Wu J Li JQ. Effect of digital economy on large-scale pig farming: an empirical study from China. Cogent Food Agric. (2023) 9:2238985. doi: 10.1080/23311932.2023.2238985

[5] Ge X Apurbo S Lu Q Zhang SX Md AR Tan YF. The impact of the epidemic experience on the recovery of production of pig farmers after the outbreak-evidence from the impact of African swine fever in Chinese pig farming. Prev Vet Med. (2022) 199:105568. doi: 10.1016/j.prevetmed.2022.105568 35008013

[6] Luo QF Liu PF Li Z. The influence of African swine fever information on consumers' preference of pork attributes and pork purchase. Can J Agric Econ. (2023) 71:49–68. doi: 10.1111/cjag.12324

[7] Jia YF Sun WS Su GF Hua JG He ZJ. The threshold effect of swine epidemics on the pig supply in China. Animals. (2022) 12:2595. doi: 10.3390/ani12192595, PMID: 36230336 PMC9558980

[8] Wang JJ Wang GY Cui YN Zhang J. How does imported pork regulate the supply and demand of China's pig market during the epidemic?: based on the analysis of African swine fever and COVID-19. Front Vet Sci. (2022) 9:1028460. doi: 10.3389/fvets.2022.1028460, PMID: 36504846 PMC9730240

[9] Tian WY Yu H Wu XM. Calculation of appropriate pig farming scale from the perspective of pollution control costs: based on the investigation of large counties in Sichuan province that export pigs. Rural Econ. (2019) 3:122–7.

[10] Wang XH Wang GY. Research on the stabilizing effect of capital breeding on pig prices. Prices Mon. (2018) 6:30–4. doi: 10.14076/j.issn.1006-2025.2018.06.06

[11] Zhang ZJ Chi L Wu JZ Zhu MS Shen C. Industrial clustering and environmental pollution: spatial-temporal dynamics and driving factors of China's feed processing industry. J Clean Prod. (2025) 498:145173. doi: 10.1016/j.jclepro.2025.145173

[12] Zhang ZW Zhou K Li JQ. Effect of labor opportunity cost on hog production fluctuation: an empirical study based on China. Cogent Food Agric. (2025) 11:2445762. doi: 10.1080/23311932.2024.2445762

[13] Breustedt G Glauben T. Driving forces behind exiting from farming in western Europe. J Agric Econ. (2007) 58:115–27. doi: 10.1111/j.1477-9552.2007.00082.x

[14] Shen YS Wu JX. Analysis of the development trend and motivations of large-scale pig farming in China. Chin J Anim Sci. (2011) 22:49-52+70.

[15] Liu F Yuan YY Zhang SS He ZW. Research on the production recovery behaviors of Chinese pig farmers under the impact of African swine fever epidemic: based on the survey of affected farmers in 21 provinces. J Agrotech Econ. (2024) 3:35–51. doi: 10.13246/j.cnki.jae.2024.03.002

[16] Li JJ Li JY Liu YK. Thoughts on inviting large capital into the agricultural sector. J Hebei Univ Econ Bus (Compr Ed). (2013) 1:77–79+95. doi: 10.14178/j.cnki.issn1673-1573.2013.01.022

[17] Kivumbi CC Yona C Hakizimana JN Misinzo G. An assessment of the epidemiology and socioeconomic impact of the 2019 African swine fever outbreak in Ngara district, western Tanzania. Vet Anim Sci. (2021) 14:100198. doi: 10.1016/j.vas.2021.100198, PMID: 34585020 PMC8455476

[18] Piao SY Jin XJ Hu SY Lee JY. The impact of African swine fever on the efficiency of China's pig farming industry. Sustainability. (2024) 16:7819. doi: 10.3390/su16177819

[19] Lu G Pan JL Zhang GH. African swine fever virus in Asia: its rapid spread and potential threat to unaffected countries. J Infect. (2020) 80:365–7. doi: 10.1016/j.jinf.2019.11.011 31758954

[20] Ma ML Wang HH Hua YZ Qin F Yang J. African swine fever in China: impacts, responses, and policy implications. Food Policy. (2021) 102:102065. doi: 10.1016/j.foodpol.2021.102065

[21] Chen H Mao L Zhang YH. Impacts of information about COVID-19 on pig farmers’ production willingness and behavior: evidence from China. J Integr Agric. (2024) 23:1429–41. doi: 10.1016/j.jia.2023.11.034

[22] Xie YH Yang YM Xie YZ. The impact of industrial agglomeration on new quality productive forces enhancement in China's pig farming industry. Sci Rep. (2025) 15:18591. doi: 10.1038/s41598-025-03461-2, PMID: 40425753 PMC12117122

[23] Bai YY Zhai YJ Zhang TZ Ren K Jia YK Zhou XY. Sustainable assessment and resource recycling opportunities identification for China's pig industry: integrating environmental, economic and social perspectives. Sustain Prod Consum. (2023) 39:425–37. doi: 10.1016/j.spc.2023.05.018

[24] Li WJ Li YJ Li TT Zhu XK. How does government support affect seed enterprises’ technological innovation performance?: an analysis based on policy, organization and market heterogeneity. Chin. Rural Econ. (2019) 9:104–23. doi: 10.20077/j.cnki.11-1262/f.2019.09.007

[25] Liu HY. Recovering farming supply chains from animal epidemics via government subsidies. Computers Ind Eng. (2024) 190:110024. doi: 10.1016/j.cie.2024.110024

[26] Wang YT Yan JZ Wu Y. Impact of policy measures on smallholders’ livelihood resilience: evidence from Hehuang Valley, Tibetan plateau. Ecol Indic. (2024) 158:111351. doi: 10.1016/j.ecolind.2023.111351

[27] Zhong FL Liu YS Ma YL Li YL He M. The government’s impact on the transformation of rural livelihoods in agropastoral regions: aquantitative analysis of farmers’ perceptions of public services in Inner Mongolia, China. Land Use Policy. (2025) 157:107642. doi: 10.1016/j.landusepol.2025.107642

[28] Hao W Shi ZZ Yang JQ Hu XD. The impact of the African swine fever epidemic on the meat consumption of Chinese residents. Res Agric Modern. (2023) 1:142–52. doi: 10.13872/j.1000-0275.2023.0017

[29] Wu Q Sun JY Chen JL. The carbon emission reduction effect and its mechanism of technological progress in the livestock industry: based on the examination of mediating effect, moderating effect and spatial spillover effect. J Technol Econ. (2023) 11:62–74.

[30] Li XL. Research on the impact of urbanization on the integration of rural industries: an analysis of the threshold effect of fiscal support for agriculture. Agric Econ Manag. (2021) 2:32–42.

[31] Lu YF Jia JF Yang Q. The impact of targeted price subsidy reform on farmers' soybean production: an empirical analysis based on the difference-in-differences method. J Agrotech Econ. (2023) 8:66–81. doi: 10.13246/j.cnki.jae.2023.08.005

[32] Lu Y Xie H Xu C. Telecommunication externality on migration: evidence from Chinese villages. China Econ Rev. (2016) 39:77–90. doi: 10.1016/j.chieco.2016.03.007

[33] Li QN Li GC. The impact of internet development on the growth of agricultural total factor productivity. J Huazhong Agric Univ (Soc Sci Ed). (2020) 4:71–78+177. doi: 10.13300/j.cnki.hnwkxb.2020.04.008

[34] Li GC. Capital deepening, land-product ratio and the growth of China's agricultural productivity: a production function analysis framework. Chin Rural Econ. (2015) 1:14–30+72. doi: 10.20077/j.cnki.11-1262/f.2015.01.002

[35] Porter ME. The Competitive Advantage of Nations. New York: The Free Press (1990).

